# Phenomenal awareness can emerge without attention

**DOI:** 10.3389/fnhum.2013.00891

**Published:** 2013-12-20

**Authors:** Jaan Aru, Talis Bachmann

**Affiliations:** ^1^Department Neurophysiology, Max-Planck Institute for Brain ResearchFrankfurt, Germany; ^2^Department of Criminal Law, Criminology and Cognitive Psychology, Laboratory of Cognitive Neuroscience, Institute of Public Law, University of TartuTallinn, Estonia

**Keywords:** attention, consciousness, awareness, afterimages, dreams, transcranial magnetic stimulation, phenomenal consciousness

In a recent debate, the views that top-down attention is necessary for consciousness (Cohen et al., 2012a,b) and that consciousness is independent of top-down attention (Tsuchiya et al., 2012) have clashed. Here, we list the overlooked or ignored arguments that should be considered before deciding that consciousness is inevitably the result of attention.

The issue of relation of *bottom-up* attention and consciousness seems to have two possible solutions according to the evidence available at present. First, based on the current evidence it may be agreed upon that conscious experience cannot be dissociated from bottom-up exogenous attention (Tsuchiya et al., 2012). Second, it is possible that there is phenomenal consciousness emerging without bottom-up attention, but empirical evidence for this is either lacking or too much controversial at present. Here, we consider some instances where simple conscious experience, i.e., phenomenal awareness emerges without *top-down* attentional deployment:

1. Sensory experiences can be brought about artificially by brain stimulation such as when using transcranial magnetic stimulation (TMS). Visual scotomas (Murd et al., 2010) or phosphenes as “visual echoes” (Jolij and Lamme, 2010) generated by early visual cortex stimulation serve as an example for simple conscious experiences evoked by TMS. Attention is not needed for these conscious experiences. Such artificial experience can be evoked at an unexpected moment and location and will become available for attention only subsequently. It could be argued that in the task where subjects evaluate their phosphenes or scotomas the subjects are still attending to these conscious phenomena. However, we claim that if a subject is performing an attending task unrelated to TMS and visual perception, but is unexpectedly given a single TMS pulse which elicits a phosphene, this phosphene will be consciously perceived.
2. Similarly, involuntary hallucinations and pathological sensations emerge without any attention to these contents. For example consider tinnitus or auditory verbal hallucinations. It would be a simple remedy for the patients if simply not attending to the hallucinations would make them disappear. Many such hallucinations come and go without the subject having any control of their duration or onset (Sacks, 2012).
3. No attention is required for dream episodes. Dreams are conscious experiences which are generally characterized by the lack of top-down attention (Hobson, 2002). The loss of top-down control and attention in typical everyday dreams is also evidenced by the peculiar feeling that accompanies lucid dreaming, where due to training some top-down control over the dream content becomes available for the subject (e.g., Voss et al., 2009).
4. Experiments and phenomenology on microgenetic formation of perceptual images and visual immediate (iconic) memory demonstrate that phenomenal-perceptual proto-objects or scenes can precede attentional selection (Bachmann, 2000; Lamme, 2004, 2010) and that non-attended objects are consciously experienced (Vandenbroucke et al., 2012).
  These arguments show that simple conscious experience could emerge independently of attention. One could argue that in all these cases even when the conscious experience is very simple (as in the case of phosphenes) the stimulus is first represented in the brain, then amplified by top-down attention and only then emerges in awareness. Thus, one could claim that these arguments are not sufficient for showing that simple conscious experience can emerge without attention. However, even if one would agree that the evidence does not convincingly demonstrate the independence of attention and consciousness, it would not imply that attention is necessary for consciousness. Furthermore, there are some additional arguments to support the independence of attention and consciousness.
5. If attention is necessary for conscious perception, attending to certain phenomenal content should not be unfavorable for experiencing this content. However, several recent studies have shown that attention has a detrimental effect on the duration of afterimages or sensory aftereffects (Lou, 2001; Bachmann and Murd, 2010; van Boxtel et al., 2010). If attention readily eliminates phenomenal experience then the awareness-related mechanisms whose activity is suppressed together with phenomenal experience must be independent from the attentional mechanisms.
6. Attending to the contents of the currently dominating image in binocular rivalry does not preclude that the competing alternative might spontaneously emerge in phenomenal awareness. If attention is necessary for consciousness and it is deployed to the dominating image, how could the suppressed image access awareness?
7. Many authors agree that consciousness and attention have different neurobiological mechanisms (Lamme, 2004; Koch and Tsuchiya, 2007; Bachmann, 2011). This of course does not deny interaction between these mechanisms in typical cases where attention has a supportive role in bringing perceptual contents to consciousness, but the independence of the neurobiological mechanisms supports the view that attention and consciousness are autonomous processes.

Finally, it is not true that in order to support the independence of consciousness and attention one has to show that attention is not needed for the stimulus to enter consciousness across a set of experimental paradigms (Cohen et al., 2012a). In fact, one could turn the argument around: if it is consistently shown by many research groups that attention is not needed for conscious perception even in one single paradigm, then this already implies that attention is not necessary for consciousness. It was also argued that the absence of the attentional effect on consciousness is a null finding which should be treated with caution (Cohen et al., 2012a). However, if this null finding is consistent over several experiments with sufficient statistical power, this result is solid by any scientific standard.

Taken together, these arguments support the view that attention and consciousness are independent from each other, but can interact. Phenomenal consciousness can emerge without attention.
